# Stroke Correlates in Chagasic and Non-Chagasic Cardiomyopathies

**DOI:** 10.1371/journal.pone.0035116

**Published:** 2012-04-16

**Authors:** José Alberto Martins da Matta, Roque Aras, Cristiano Ricardo Bastos de Macedo, Cristiano Gonçalves da Cruz, Eduardo Martins Netto

**Affiliations:** 1 Hospital Universitário Professor Edgard Santos, Universidade Federal da Bahia, Salvador, Bahia, Brazil; 2 Hospital Ana Nery, Universidade Federal da Bahia, Salvador, Bahia, Brazil; 3 Hospital Universitário Professor Edgard Santos, Universidade Federal da Bahia, Salvador, Bahia, Brazil; Université Joseph Fourier, France

## Abstract

**Background:**

Aging and migration have brought changes to the epidemiology and stroke has been shown to be independently associated with Chagas disease. We studied stroke correlates in cardiomyopathy patients with focus on the chagasic etiology.

**Methodology/Principal Findings:**

We performed a cross-sectional review of medical records of 790 patients with a cardiomyopathy. Patients with chagasic (329) and non-chagasic (461) cardiomyopathies were compared. There were 108 stroke cases, significantly more frequent in the Chagas group (17.3% versus 11.1%; p<0.01). Chagasic etiology (odds ratio [OR], 1.79), pacemaker (OR, 2.49), atrial fibrillation (OR, 3.03) and coronary artery disease (OR, 1.92) were stroke predictors in a multivariable analysis of the entire cohort. In a second step, the population was split into those with or without a Chagas-related cardiomyopathy. Univariable post-stratification stroke predictors in the Chagas cohort were pacemaker (OR, 2.73), and coronary artery disease (CAD) (OR, 2.58); while atrial fibrillation (OR, 2.98), age over 55 (OR, 2.92), hypertension (OR, 2.62) and coronary artery disease (OR, 1.94) did so in the non-Chagas cohort. Chagasic stroke patients presented a very high frequency of individuals without any vascular risk factors (40.4%; OR, 4.8). In a post-stratification logistic regression model, stroke remained associated with pacemaker (OR, 2.72) and coronary artery disease (OR, 2.60) in 322 chagasic patients, and with age over 55 (OR, 2.38), atrial fibrillation (OR 3.25) and hypertension (OR 2.12; *p* = 0.052) in 444 non-chagasic patients.

**Conclusions/Significance:**

Chagas cardiomyopathy presented both a higher frequency of stroke and an independent association with it. There was a high frequency of strokes without any vascular risk factors in the Chagas as opposed to the non-Chagas cohort. Pacemaker rhythm and CAD were independently associated with stroke in the Chagas group while age over 55 years, hypertension and atrial fibrillation did so in the non-Chagas cardiomyopathies.

## Introduction

Chagas disease (CD) is caused by the flagellate protozoan Trypanosoma cruzi and its main mechanism of transmission is transcutaneous inoculation of the parasite by excreta of infected insects [1]–[3]. Infection with T cruzi is an enzootic disease, which can lead to human disease when the insect vectors—triatomine bugs—reach their domestic cycle by infesting human mud houses in poor communities. In non-endemic countries, blood transfusion, organ transplantation, and vertical transmission are more likely routes of infection [1], [3]–[6]. Oral transmission, common in the enzootic cycle [6], has only recently been reported in humans [7], [8]. An estimated 10 million people are infected worldwide, mostly in Latin America and more than 25 million are at infection risk [9]–[11]. Its annual death toll is estimated to claim 10,000 lives [11], [12] and the 10-year mortality rate may range from 9% to 85%, depending on clinical markers of cardiac damage [13]. Although vector transmission has been virtually interrupted in most endemic countries [9], non-permanent sanitation control [9], [14], [15], alternative transmission routes [7], [8], escalating migration [4], [12], and the increase in life expectancy have resulted in a pressing pattern of Chagas cardiomyopathy on the overall burden of chronic disease [4], [16]–[18]. Nonetheless, Chagas disease is still posted among the 20 most important neglected tropical diseases [12].

There are two successive stages, an acute and a chronic phase. The acute phase lasts 6 to 8 weeks, with spontaneous recovery in more than 95% of patients. In the first years of the chronic phase, most infected patients have no clinical evidence of an ongoing illness. This stage of the chronic phase is called the indeterminate form and in most patients it persists indefinitely [1]–[3], [5], [6]. Ten to thirty years after it started, up to 30% of individuals will present organ damage mainly affecting the heart muscle, leading to Chagas cardiomyopathy which may result in severe cardiac dilatation and contractile dysfunction [3]–[6], mostly due to a parasite-triggered immune-mediated inflammation [19]. Patients will often present malignant arrhythmias, heart blocks or cardiogenic embolism; the latter one a common cause of ischemic stroke [3], [5], [6]. Likewise, stroke carries significant morbidity and lethality [4], [20], [21], is often undertreated and is even considered the most neglected aspect in the management of chagasic cardiomyopathy [5].

Although risk factors for ischemic stroke are reasonably determined for many cardiac structural diseases, including the dilated non-chagasic cardiomyopathies [21]–[23], they still have not been properly established for patients with the chagasic cardiomyopathy [5]. Few observational studies have been done on risk correlates for stroke in Chagas cardiomyopathy, most have focused on CD as a risk factor without establishing a differentiation between CD itself and the chagasic cardiomyopathy and present significant heterogeneity in sampling and methods [24]–[29]. Only one study was performed specifically to determine the risk factors for stroke in chagasic patients who already presented with a cardiomyopathy [30]. The purpose of this study was to describe the frequency of stroke correlates in a population presenting with a cardiomyopathy, representative of a tertiary center in northeastern Brazil with a specific focus on the chagasic etiology to define its clinical profile according to stroke risk.

## Methods

### Ethics Statement

The Institutional Ethics Board of the Hospital Universitario Professor Edgard Santos at the Federal University of Bahia approved the study. The data were analyzed anonymously and the Institutional Ethics Board waived consent.

The medical records of 806 non–selected consecutive patients who demanded care between September/2003 and June/2008 in a hospital-based outpatient cardiomyopathy clinic in the northeastern Brazilian state of Bahia were reviewed in this cross-sectional single-center study. A diagnosis of cardiomyopathy was a pre-requisite for admission and follow-up in this Clinic.

This specialized clinic utilizes a standardized protocol as a medical record for routine patient care, which facilitates clinical follow-up and allows for straightforward data retrieval whenever required. This protocol covers full medical history and physical examination, through the traditional systematic description of symptoms and a structured yes/no (numeric) or quantitative design. This section includes, among others, specific items on dyspnea, edema, alcoholism, smoking status, hypertension, diabetes mellitus, chronic renal failure, coronary artery bypass graft (CABG) or percutaneous transluminal coronary angioplasty (PTCA), coronary artery disease, stroke, permanent pacemaker and cardioversion. Chagas serologic status, the regimen and adherence to drug treatments, a complete physical examination, detailed data on the electrocardiogram (ECG) and basal rhythm, Holter monitoring, chest x-ray, echocardiogram, myocardial stress scyntigraphy, cinecoronariography, blood chemistry and other laboratory procedures are also included in this form. Hospital admission and a non-scheduled visit to the clinic or emergency room are specified by the precipitating event as stroke, resuscitated cardiopulmonary arrest or cardioversion-requiring ventricular tachycardia, pulmonary embolism, decompensated heart failure, acute pulmonary edema, cardiac surgery, and any other verified conditions, if there is a diagnosis. Conclusion sections include information on the presence of clinical heart failure, functional class and the most likely etiologic diagnosis with certification of review by the medical staff.

A diagnosis of Chagas disease (CD) required a positive serologic status that was defined by any combination of at least two positive serologic tests for CD (indirect immunofluorescence, indirect hemagglutination, or enzyme-linked immunosorbent assay) at data collection (31;32), At Chart review, demographic, clinical data, and available complementary diagnostic work-up were compliant with the accepted published evidence [21], [31]–[34].

A cardiomyopathy was defined as a structural disease of the myocardium associated with cardiac dysfunction. They were initially classified as dilated cardiomyopathy or a form of non-dilated cardiomyopathy (hypertrophic, restrictive and arrhythmogenic right ventricular dysplasia) [34]. Dilated cardiomyopathy was characterized by dilatation and impaired contraction of the left ventricle or both ventricles; idiopathic, secondary to a specific etiology or associated with a recognized cardiovascular disease in which the degree of myocardial dysfunction was not explained by the abnormal loading conditions or the extent of ischemic damage [34].

Right bundle branch block, >1 ventricular premature beats (VPB's) by ECG either polymorphous or repetitive, non-sustained ventricular tachycardia, 2^nd^ and 3^rd^ degree atrio-ventricular block, sinus bradycardia <40 beats/min, sinus node dysfunction, left bundle branch block, atrial fibrillation, inactive segment and primary alterations of ST-T wave are typical ECG changes in Chagas cardiomyopathy by a Brazilian Expert Consensus [31]. Sinus bradycardia >40 beats/min, lone left anterior hemiblock, non-specific ST-T changes, incomplete right bundle branch block, 1^st^ degree atrio-ventricular block and isolated VPB's are non-specific ECG changes by this Consensus.

Chagas cardiomyopathy was diagnosed only in patients presenting a positive serologic status and one of the two following characteristics: either typical ECG changes independently of the presence of symptoms or nonspecific ECG changes plus typical symptoms including a dilated heart with systolic dysfunction [31], [32]. Patients fulfilling the above criteria formed a Chagas cardiomyopathy group and the remaining patients formed the non-Chagas cardiomyopathy group.

Stroke was defined as a focal neurological impairment of sudden onset, lasting more than 24 hours and of presumed vascular origin, excluding symptoms associated with epidural and subdural hemorrhage, poisoning, and those caused by trauma. A history of global impairment or deep coma (subarachnoid hemorrhage or coma of systemic vascular origin) was also an exclusion criterion, since the outcome of interest was ischemic stroke. This was adapted from the standard definition of the World Health Organization that takes stroke as a clinically defined disease, irrespective of access to technological equipments [33].

Clinical correlates were sorted based on prevalence, clinical relevance, and availability. Age 55 was taken as an overall cut point for vascular risk; current smokers were those who smoked for over a year in their lifetimes and were still smoking; and current alcohol overuse required drinking of more than five doses of a distilled beverage per week at the time of evaluation. As parameters for heart failure, the variables dyspnea and edema were entered if there were shortness of breath at rest or upon exertion and presence of dependent edema of the lower limbs, respectively. Left ventricular dysfunction was categorized as an ejection fraction below 45% in the echocardiogram. The variable pacemaker referred to a permanent pacing device previously implanted, cardioversion stood for a history of either resuscitated cardiac arrest or cardioversion requiring ventricular tachycardia, and any basal rhythm other than normal sinus was described as a rhythm non-sinus, while other variables listed were self-explanatory.

### Statistics

The unpaired t-test was used for continuous variables and Pearson chi-square or Fisher's exact test for categorical variables, with *p*<0.05 considered significant. A case-wise deletion was adopted for the missing values at univariable analysis and a listwise deletion for multivariable analysis. Baseline variables were presented and compared between patients with or without Chagas disease. Stroke correlates were compared between patients with or without a stroke and variables with *p*<0.05, plus sex and hypertension that is commonly associated with stroke in the literature besides being highly frequent in our sample, were further analyzed trough a logistic regression model with stroke as a dependent variable. Next, we performed a post-stratification analysis according to the etiology of the cardiomyopathy, as either chagasic or non-chagasic. Only variables associated at the previous step plus age, sex and hypertension were stratified. Further, we took only variables with p<0,05 plus age and sex and applied a dichotomous logistic regression model taking stroke as a dependent variable. The statistical package for the social sciences (SPSS 17.0 Inc., Chicago, Illinois) was employed.

## Results

Eight hundred and six patients were seen in the Clinic through this period. Information about a stroke diagnosis was missing for 16 individuals, leaving 790 available for analysis. Patients included were 58.3+−13.2 years old (437 [55.3%] males), 461 (58.4%) presented a non Chagas-related cardiomyopathy and 329 (41.6%) a Chagas-related cardiomyopathy. Within the non-Chagas group, 433 (54.8%) exhibited a dilated-type cardiomyopathy and 28 (3.5%) evidenced a form of non-dilated cardiomyopathy, either restrictive or hypertrophic.

Since cases with missing information were excluded from analysis through a variable-wise deletion, their number varied down the column of clinical and demographic characteristics in Table 1. The prevalences of clinical and demographic characteristics (Table 1), both in the full cohort and in the split cohorts, whether patients presented with or without a Chagas-related cardiomyopathy, are also shown. There were 108 (13.7%) stroke cases, 57 in the Chagas cohort and 51 in the non-Chagas cohort. Vascular risk factors; smoking (*p* = 0,005), hypertension (*p*<0.01), diabetes (*p* = 0.09), CABG or PTCA (*p* = 0.001), coronary artery disease (*p*<0.01) and chronic renal failure (*p*<0.04) exhibited higher prevalences in the non-Chagas cohort. However, stroke was more commonly seen in the Chagas cohort (17.3%) than in the non-Chagas cohort (11.1%; *p* = 0.01). Still, the prevalences of pacemaker and rhythm non-sinus were significantly higher (*p* = 0.01) in the Chagas cohort.

**Table 1:** Baseline Characteristics of 790 Cardiomyopathy Patients.

Clinical and demographic characteristics	Cases available		Chagas disease		*p* value
	n	%	Yes	No	
			(n = 329 [41.6%])	(n = 461 [58.4%])	
Age (mean [SD])	789	58.3 [13.2]	58.8 [11.4]	58.0 [14.3]	0.409
Age over 55 years	789	62.0	63.5	60.9	0.449
Male sex	790	55.3	53.5	56.6	0,384
Non-White	773	82.4	85.8	80.0	0,038
Alcohol abuse	779	7.3	5.8	8.4	0.176
Current smoker	790	15.6	11.2	18.7	0.005
Hypertension	789	54.4	42.1	63.1	0.000
Diabetes mellitus	789	11.8	9.5	13.4	0.086
Stroke	790	13.7	17.3	11.1	0.012
Pacemaker	789	8.2	15.2	3.3	<0.001
Cardioversion^a^	789	2.5	2.7	2.4	0.753
Rhythm non-sinus	789	27.0	35.6	20.9	0.000
Atrial fibrillation	789	8.2	10.0	7.0	0.122
LV dysfunction^b^	621	66.8	71.1	63.7	0.530
Dyspnea	789	86.3	86.0	86.6	0.817
Edema	778	60.8	62.7	59.5	0.370
Left heart thrombus	599	2.8	2.9	2.8	0.936
CABG or PTCA^c^	776	4.1	1.2	6.2	0.001
Coronary artery disease	772	16.1	7.4	22.4	<0.001
Chronic renal failure	785	6.5	4.3	8.0	0.036

SD: standard deviation.

a Cardioversion for ventricular tachycardia or cardiac arrest.

b Left ventricular ejection fraction below 45%.

c Coronary artery bypass graft or percutaneous transluminal coronary angioplasty.

Univariable correlates associated with stroke in the entire cohort (Table 2) were age over 55 years, hypertension (marginally, p = 0,054), chagasic etiology, pacemaker, cardioversion, rhythm non-sinus, atrial fibrillation and coronary artery disease. After adjustment through multivariable analysis, chagasic etiology (OR 1.79), pacemaker (OR, 2.49), atrial fibrillation (OR, 3.03) and coronary artery disease (OR, 1.92) persisted significantly associated with stroke. Left ventricular ejection fraction<45%, dyspnea and edema were not associated with stroke.

**Table 2:** Clinical and Demographic Correlates of Stroke in 790 Cardiomyopathy Patients.

Clinical and demographic correlates	Cases available (n)	Stroke (%):		Unadjusted *p* value^a^	Adjusted odds ratio^b^	95% confidence interval
		YES	NO			
Age over 55	789	72.2	60.4	0.018	1.41	0.87–2.27
Male sex	790	52.8	55.7	0.568	0.98	0.64–1.50
Non-white	773	87.6	81.6	0.131	…	…
Alcohol abuse	779	6.5	7.5	0.719	…	…
Current smoker	790	12.0	16.1	0,276	…	…
Hypertension	789	63.0	53.0	0,054	0.69	0.43–1.10
Diabetes mellitus	789	13.9	11.5	0.466	…	…
Chagasic etiology	790	52.8	39.9	0,012	1.79	1.13–2.84
Pacemaker	789	15.7	7.0	0,002	2.49	1.08–5.77
Cardioversion^c^	789	6.5	1.9	0,005	2.26	0.80–6.43
Rhythm non-sinus	789	36.1	25.6	0,022	0.78	0.38–1.60
Atrial fibrillation	789	15.7	7.0	0,002	3.03	1.33–6.94
LV dysfunction^d^	621	69.8	66.4	0.533	…	…
Dyspnea	789	84.3	86.6	0.504	…	…
Edema	778	69.2	59.5	0,056	…	…
Left heart thrombus	599	1.3	3.1	0.392	…	…
CABG or PTCA^e^	776	0.9	4.6	0.110	…	…
Coronary artery disease	772	23.4	14.9	0,027	1.92	1.12–3.31
Chronic renal failure	785	9.3	6.1	0.210	…	…

a Unadjusted results based on Pearson chi-square or Fisher's exact test as appropriate.

b Adjusted results based on logistic regression, including all variables with unadjusted *p*<0.05. Missing cases were 22 due to listwise deletion. 768 patients remained in the analysis, 107 with a stroke and 661 without a stroke.

c Cardioversion for ventricular tachycardia or cardiac arrest.

d Left ventricular ejection fraction below 45%.

e Coronary artery bypass graft or percutaneous transluminal coronary angioplasty.

In a second step, the population was split into two groups, those without (Table 3) or with (Table 4) a Chagas related-cardiomyopathy. Univariable post-stratification stroke predictors in the Chagas cohort (Table 4) were pacemaker (OR, 2.73; 95% CI, 1.38–5.39),and coronary artery disease (OR 2.58; 95% CI, 1.05–6.36). In the non-Chagas cohort (Table 3), age over 55 (OR, 2.92; 95% CI, 1.42–5.98), atrial fibrillation (OR, 2.98; 95% CI, 1.26–7.05), hypertension (OR, 2.62; 95% CI 1.28–5.39) and coronary artery disease were (OR, 1.94; 95% CI, 1.03–3.66) were the post-stratification univariable predictors. Vascular risk factors (VRFs), smoking, hypertension, diabetes, CABG or PTCA, coronary artery disease, and chronic renal failure were computed into a single composite co-variable (VRFco). At an univariable analysis, there was a high frequency of chagasic stroke patients without VRFco (23 out of 56 / 40,4%) and this was clearly distinct from non-chagasic stroke patients (6 out of 49/ 12,2%; p<0,001).

**Table 3:** Stroke Correlates in Non-Chagasic Patients.

Clinical correlates	Stroke (n)				Unadjusted odds ratio^a^	95% confidence interval	Adjusted odds ratio^b^	95% confidence interval
	Yes (51)		No (410)					
	n	%	n	%				
**Age over 55 years**	41	80.4	239	58.4	2.92	1.42–5.98	2.38	1.13–5.00
**Male Sex**	29	56.9	232	56.6	0.99	0.55–1.78	1.00	0.54–1.85
**Pacemaker**	1	2.0	14	3.4	0.56	0.07–4.38	…	…
**Atrial fibrilation**	8	15.7	24	5.9	2.98	1.26–7.05	3.25	1.32–7.96
**Hypertension**	41	80.4	250	61.0	2.62	1.28–5.39	2.12	0.99–4.51
**CAD**	17	34.0	83	21.0	1.94	1.03–3.66	1.50	0.77–2.92

a Unadjusted results based on Pearson chi-square or Fisher's exact test as appropriate. See text for explanation on how variables were previously selected. All 461 non-chagasic patients were included in the analysis.

b Adjusted results based on logistic regression, all listed variables included. See text for explanation on how variables were previously selected. Missing cases were 17 due to listwise deletion. 444 patients remained in the analysis, 50 with a stroke and 394 without a stroke.

CAD: Coronary artery disease.

**Table 4:** Stroke Correlates in Chagasic Patients.

Clinical correlates	Stroke (n)				Unadjusted odds ratio^a^	95% confidence interval	Adjusted odds ratio^b^	95% confidence interval
	Yes (57)		No (272)					
	n	%	n	%				
**Age over 55 years**	37	64.9	172	63.2	1.08	0.59–1.95	0.97	0.52–1.79
**Male Sex**	28	49.1	148	54.4	1.36	0.70–2.19	0.83	0.46–1.50
**Pacemaker**	16	28.1	34	12.5	2.73	1.38–5.39	2.72	1.37–5.40
**Atrial fibrilation**	9	15.8	24	8.8	1.94	0.85–4.43	…	…
**Hypertension**	27	47.4	111	41.0	1.30	0.73–2.30	…	…
**CAD**	8	14.0	16	5.9	2.58	1.05–6.36	2.60	1.03–6.56

a Unadjusted results based on Pearson chi-square or Fisher's exact test as appropriate. See text for explanation on how variables were previously selected. All 329 chagasic patients were included in the analysis.

b Adjusted results based on logistic regression, all listed variables included. See text for explanation on how variables were previously selected. Missing cases were 7 due to listwise deletion. 322 patients remained tin the analysis, 54 with a stroke and 268 without a stroke.

CAD: Coronary artery disease.

Taking patients without (Table 3) or with a Chagas related-cardiomyopathy (Table 4) and further entering those data on a dichotomous logistic regression model for multivariable adjustment, stroke remained associated with age over 55 years (OR 2.38; 95% CI, 1.13–5.00), atrial fibrillation (OR 3.25; 95% CI, 1.32–7.96) and marginally with hypertension (OR 2.12; *p* = 0,052; 95% CI, 0.99–4.51) in the 444 non-chagasic patients (Table 3). Remaining predictors of stroke for the 322 chagasic patients (Table 4) were pacemaker (OR = 2.72; 95%CI: 1.37–5.40) and coronary artery disease (OR = 2.60; *p* = 0.043; 95%CI:1.03–6.56). Due to a variable number of cases available for each co-variable in the logistic regression model, there were 17 missing cases in the non-Chagas cohort and 7 in the Chagas cohort, remaining 104 patients with a stroke, 50 and 54 for each cohort respectively.

None of the pacemaker recipients presented concomitant atrial fibrillation in the entire cohort. All patients with atrial fibrillation were put on international normalized ratio (INR) adjusted long-term warfarin treatment. Since warfarin treatment was more likely a consequence than a cause of stroke in this population, or otherwise it was prescribed to patients with classical risk factors such as atrial fibrillation and heart failure combined, we did not include it in the final analysis, as we are searching for additional non-established risk factors for stroke in Chagas cardiomyopathy.

## Discussion

Chagas disease and its related cardiomyopathy have long been associated with thrombo-embolism and stroke in necropsy studies [20], [35], [36]. Nonetheless, most clinical series have only been reported in the last decade [24]–[30].

Apart from all previous reports, our study compares the cross-sectional frequency of stroke and its correlates in patients with and without a Chagas-related cardiomyopathy in a hospital-based cardiomyopathy cohort. Our patients were predominantly over 55 years of age, male sex, non-white, with a high frequency of stroke (108/790 = 13.7%) and Chagas cardiomyopathy (329/790 = 41.6%). While stroke was more prevalent in the Chagas cohort as compared to the non-Chagas cohort, there was a much higher frequency of chagasic stroke patients without vascular risk factors when compared to the non-Chagas cohort. Though we came along with a singular design, our general findings are consistent with at least two previous studies from Carod-Artal which reported on Chagas disease and stroke in the emergency department [24], [25].

The core findings of our study, however, derive from an additional two-step multivariable analysis. In the first step, enclosing the entire cohort, Chagas etiology (OR = 1.79), pacemaker (OR = 2.49), atrial fibrillation (OR = 3.03), and coronary artery disease (OR = 1.92) were independently associated with stroke. In the second step, a post-stratification dichotomous logistic regression model was applied to the cohort, by splitting it into Chagas and non-Chagas groups. In the Chagas group, pacemaker (OR 2.72) and coronary artery disease (OR = 2.60) remained as independent predictors Though such association between stroke and pacemaker rhythm has not been clearly reported, during recent years, when most clinical series concerning Chagas disease and stroke emerged [24]–[30], some of the data pointed in the same direction. Carod-Artal showed an association for pacemaker rhythm and chagasic stroke, though with a much smaller number of cases [25]. Whether a pacemaker rhythm itself may cause dissinchrony and bring about stroke or stands as a surrogate marker for other clinical variables, it was not possible to evaluate in this study. While the association of pacemaker use in Chagas' patients with stroke even after adjustment is interesting, this finding needs both confirmation from prospective studies and clarifying how pacemaker users differ from non-users. Still, since the pathogenesis of CD itself makes it highly prone to embolism [5], [19], it may be hard to elicit supervening risk factors for cardiogenic embolism.

On the other hand, some of the classical stroke risk factors, were independent predictors in the non-Chagas group, namely age over 55 years (OR 2.38), atrial fibrillation (OR 3.25) and albeit marginally, hypertension (OR 2.12; *p* = 0,052). Noteworthy, the strength of the association was particularly strong for atrial fibrillation, post stratification, in the non-Chagas group. While atrial fibrillation (AF) was not associated with stroke in the Chagas cohort (unadjusted OR 1.94; 95% CI:0.85–4.43), it is necessary to account for protection given by anticoagulation, since those patients were treated with warfarin. Souza, in a recent cohort study developed a stroke risk score for CD. However, AF could not be within the score, since none of the patients with AF, all of them on warfarin, presented a stroke during follow-up [29]. We admit that AF, a well known risk factor for heart disease and stroke, also entails risk for CD patients, yet due to its highly emboligenic pathology [20], it may not impact to the same degree as in the non-chagasic cardiomyopathies, besides the fact that the established and well validated use of warfarin precludes a clinical trial.

Although our study could not show an association of stroke with systolic dysfunction, this may be hard to demonstrate in a transversal study, due to the very biological and sequential nature of the myocardial pathology, whereas such association has already been shown in at least two cohort studies [29], [30]. Besides this, since the two groups that we compared, Chagas and non-Chagas related cardiomyopathies, are themselves associated with ventricular dysfunction and embolism, what we have actually shown was that they did not differ in this clinical aspect.

A small number of patients presented with coronary artery disease (24/7.4%) in the Chagas cohort and its presence was associated with stroke, adding further data on the changing pattern of Chagas disease, in accordance with recently reported trends [16]; [17]. Population ageing is leading to an increase in the number of older adults infected with *T. cruzi* and age-related co-morbidity is rising as most studies of the natural history of Chagas disease have centered upon younger people [3], [6].

Our study presents a number of limitations. Since our population was derived from a Clinic where a Cardiomyopathy represented a pre-requisite for admission, we have actually described stroke correlates in cardiomyopathy patients with emphasis on the Chagas-related cohort, taking the non-Chagas cohort as a control group. Though we were apt to describe how Chagas and non-Chagas related cardiomyopathies differed in stroke risk factors, we could not broadly point at stroke predictors due to lack of a non-cardiopathy control group. Whereas all information originated from a Clinic attending patients with a previously structured protocol as a medical record, data retrieval for this specific study was retrospective, followed by a cross-sectional analysis, and so it lacked statistical power for definite recommendations. Yet, since no data were collected regarding the pacemaker carriers, we could not ascertain whether the type of pacemaker played a role or how carriers differed from non-carriers in their characteristics. We are presently developing a larger sample cohort-type study in this population with focus on stroke predictors and complete clinical information on pacemaker carriers which should allow for an improved understanding about Chagas disease and stroke in that particular area.

We have identified three areas of particular interest in our study. First, not only chagasic patients presented a high prevalence of stroke (17.3%, 1 in 6 patients) but the Chagas-related cardiomyopathy was independently associated with it. Second, there was a high frequency of stroke patients in the chagasic cohort without vascular risk factors (40%, p<0.001), while just about 1 in 10 patients in the non-chagasic cohort (12%) presented a stroke without any vascular risk factors, suggesting other potential mechanisms intrinsic to Chagas cardiomyopathy.

Third, in a direct multivariable cross-sectional comparison between patients with a Chagas-related cardiomyopathy and those with non-Chagas cardiomyopathies, stroke was associated with age over 55 years, atrial fibrillation and hypertension in the non-Chagas group and just pacemaker and coronary artery disease in the Chagas group, in which a highly emboligenic pathogenesis may limit the determination of supervening stroke risk factors. We reinforce the idea that further studies are warranted in those particular areas.

## References

[1] Prata A (2001). Clinical and epidemiological aspects of Chagas disease.. Lancet Infect.

[2] Hidron A, Vogenthaler N, Santos-Preciado JI, Rodriguez-Morales AJ, Franco-Paredes C (2010). Cardiac Involvement with Parasitic Infections.. Clin Microbiol Rev.

[3] Rassi A, Rassi A, Little WC (2000). Chagas' heart disease.. Clin Cardiol.

[4] Armaganijan L, Morillo CA (2010). Chagas disease: 101 years of solitude! Time for action.. Stroke.

[5] Biolo A, Ribeiro AL, Clausell N (2010). Chagas cardiomyopathy–where do we stand after a hundred years?. Prog Cardiovasc Dis.

[6] Pinto Dias JC (1995). Natural history of Chagas disease.. Arq Bras Cardiol.

[7] Dias JP, Bastos C, Araujo E, Mascarenhas AV, Martins NE (2008). Acute Chagas disease outbreak associated with oral transmission.. Rev Soc Bras Med Trop.

[8] Valente SA, da Costa Valente V, das Neves Pinto AY, de Jesus Barbosa César M, dos Santos MP (2009). Analysis of an acute Chagas disease outbreak in the Brazilian Amazon: human cases, triatomines, reservoir mammals and parasites.. Trans R Soc Trop Med Hyg.

[9] Coura JR, Dias JC (2009). Epidemiology, control and surveillance of Chagas disease: 100 years after its discovery.. Mem Inst Oswaldo Cruz.

[10] Hotez PJ, Molyneux DH, Fenwick A, Kumaresan J, Sachs SE (2007). Control of Neglected Tropical Diseases.. N Engl J Med.

[11] World Health Organization (2010). Chagas Disease. Geneva, Switzerland. Fact sheet 340.. http://www.who.int/mediacentre/factsheets/fs340/en/index.html.

[12] World Health Organization (2010). Working to overcome the global impact of neglected tropical diseases. First WHO report on neglected tropical diseases. Geneva, Switzerland.. http://www.who.int/neglected_diseases/2010report/en/.

[13] Rassi A, Rassi A, Little WC, Xavier SS, Rassi SG (2006). Development and Validation of a Risk Score for Predicting Death in Chagas' Heart Disease.. N Engl J Med.

[14] Bastos CJC, Aras R, Mota G, Reis F, Dias JP (2010). Clinical Outcomes of Thirteen Patients with Acute Chagas Disease Acquired through Oral Transmission from Two Urban Outbreaks in Northeastern Brazil.. PLoS Negl Trop Dis.

[15] Delgado S, Castillo Neyra R, Quispe Machaca VR, Ancca Juárez J, Chou Chu L (2011). A history of chagas disease transmission, control, and re-emergence in peri-rural La Joya, Peru.. PLoS Negl Trop Dis.

[16] Lima-Costa MF, Barreto SM, Guerra HL, Firmo JO, Uchoa E (2001). Ageing with *Trypanosoma cruzi* infection in a community where the transmission has been interrupted: the Bambui Health and Ageing Study (BHAS).. International Journal of Epidemiology.

[17] Lima-Costa MF, Barreto SM, Guerra H (2002). Chagas' disease among older adults: branches or mainstream of the present burden of Trypanosoma cruzi infection?. International Journal of Epidemiology.

[18] World Health Organization (2008). Global Burden of Disease 2004 update. Geneva, Switzerland: World Health Organization.. http://www.who.int/healthinfo/global_burden_disease/2004_report_update/en/index.html.

[19] Marin-Neto JA, Cunha-Neto E, Maciel BC, Simoes MV (2007). Pathogenesis of Chronic Chagas Heart Disease.. Circulation.

[20] Aras R, da Matta JA, Mota G, Gomes I, Melo A (2003). Cerebral infarction in autopsies of chagasic patients with heart failure.. Arq Bras Cardiol.

[21] Goldstein LB, Bushnell CD, Adams RJ, Appel LJ, Braun LT (2011). Guidelines for the Primary Prevention of Stroke. A Guideline for Healthcare Professionals from the American Heart Association/American Stroke Association.. Stroke.

[22] Lip GY, Nieuwlaat R, Pisters R, Lane DA, Crijns HJ (2010). Refining clinical risk stratification for predicting stroke and thromboembolism in atrial fibrillation using a novel risk factor-based approach: the euro heart survey on atrial fibrillation.. Chest.

[23] Gage BF, van Walraven C, Pearce L, Hart RG, Koudstaal PJ (2004). Selecting Patients With Atrial Fibrillation for Anticoagulation.. Circulation.

[24] Carod-Artal FJ, Vargas AP, Melo M, Horan TA (2003). American trypanosomiasis (Chagas' disease): an unrecognised cause of stroke.. J Neurol Neurosurg Psychiatry.

[25] Carod-Artal FJ, Vargas AP, Horan TA, Nunes LG (2005). Chagasic cardiomyopathy is independently associated with ischemic stroke in Chagas disease.. Stroke.

[26] Leon-Sarmiento FE, Mendoza E, Torres-Hillera M, Pinto N, Prada J (2004). Trypanosoma cruzi-associated cerebrovascular disease: a case-control study in Eastern Colombia.. J Neurol Sci Jan.

[27] Lima Costa MF, Matos DL, Ribeiro AL (2010). Chagas Disease Predicts 10-Year Stroke Mortality in Community-Dwelling Elderly.. Stroke.

[28] Paixao LC, Ribeiro AL, Valacio RA, Teixeira AL (2009). Chagas disease: independent risk factor for stroke.. Stroke.

[29] Sousa AS, Xavier SS, Freitas GR, Hasslocher-Moreno A (2008). Prevention strategies of cardioembolic ischemic stroke in Chagas' disease.. Arq Bras Cardiol.

[30] Nunes MC, Barbosa MM, Ribeiro AL, Barbosa FB, Rocha MO (2009). Ischemic cerebrovascular events in patients with Chagas cardiomyopathy: a prospective follow-up study.. J Neurol Sci.

[31] Secretaria de Vigilância em Saúde do Ministério da Saúde (2005). Consenso Brasileiro em Doença de Chagas.. Rev Soc Bras Med Trop.

[32] Andrade JP, Marin Neto JA, Paola AA, Vilas-Boas F, Oliveira GM (2011). I Latin American Guidelines for the diagnosis and treatment of chagas' heart disease: executive summary.. Arq Bras Cardiol.

[33] World Health Organization (2005). WHO STEPS Stroke Manual: the WHO STEPwise approach to stroke surveillance. Geneva, Switzerland: WHO Press.. http://www.who.int/ncd_surveillance/en/steps_stroke_manual_v1.2.pdf.

[34] World Health Organization (1996). Report of the 1995 World Health Organization/International Society and Federation of Cardiology Task Force on the Definition and Classification of Cardiomyopathies.. Circulation.

[35] Oliveira JS, Mello De Oliveira JA, Frederigue U, Lima Filho EC (1981). Apical aneurysm of Chagas's heart disease.. Br Heart J.

[36] Samuel J, Oliveira M, Correa De Araujo RR, Navarro MA, Muccillo G (1983). Cardiac thrombosis and thromboembolism in chronic Chagas' heart disease.. Am J Cardiol.

